# Direct and Indirect Effects of Penguin Feces on Microbiomes in Antarctic Ornithogenic Soils

**DOI:** 10.3389/fmicb.2018.00552

**Published:** 2018-04-03

**Authors:** Yudong Guo, Nengfei Wang, Gaoyang Li, Gabriela Rosas, Jiaye Zang, Yue Ma, Jie Liu, Wenbing Han, Huansheng Cao

**Affiliations:** ^1^Department of Bioengineering, College of Marine Sciences and Biological Engineering, Qingdao University of Science and Technology, Qingdao, China; ^2^Key Lab of Marine Bioactive Substances, First Institute of Oceanography, State Oceanic Administration, Qingdao, China; ^3^College of Computer Science and Technology, Jilin University, Changchun, China; ^4^Center for Fundamental and Applied Microbiomics, Biodesign Institute, Arizona State University, Tempe, AZ, United States; ^5^College of Chemistry and Chemical Engineering, Qingdao University, Qingdao, China

**Keywords:** Antarctica, geochemical properties, network, penguin, ornithogenic soil, microbiome

## Abstract

Expansion of penguin activity in maritime Antarctica, under ice thaw, increases the chances of penguin feces affecting soil microbiomes. The detail of such effects begins to be revealed. By comparing soil geochemistry and microbiome composition inside (one site) and outside (three sites) of the rookery, we found significant effects of penguin feces on both. First, penguin feces change soil geochemistry, causing increased moisture content (MC) of ornithogenic soils and nutrients C, N, P, and Si in the rookery compared to non-rookery sites, but not pH. Second, penguin feces directly affect microbiome composition in the rookery, not those outside. Specifically, we found 4,364 operational taxonomical units (OTUs) in 404 genera in six main phyla: Proteobacteria, Actinobacteria, Gemmatimonadetes, Acidobacteria, Chloroflexi, and Bacteroidetes. Although the diversity is similar among the four sites, the composition is different. For example, penguin rookery has a lower abundance of Acidobacteria, Chloroflexi, and Nitrospirae but a higher abundance of Bacteroidetes, Firmicutes, and Thermomicrobia. Strikingly, the family Clostridiaceae of Firmicutes of penguin-feces origin is most abundant in the rookery than non-rookery sites with two most abundant genera, *Tissierella* and *Proteiniclasticum*. Redundancy analysis showed all measured geochemical factors are significant in structuring microbiomes, with MC showing the highest correlation. We further extracted 21 subnetworks of microbes which contain 4,318 of the 4,364 OTUs using network analysis and are closely correlated with all geochemical factors except pH. Our finding f penguin feces, directly and indirectly, affects soil microbiome suggests an important role of penguins in soil geochemistry and microbiome structure of maritime Antarctica.

## Introduction

Antarctica is unique due to its isolation from other continents, extreme climate, minimum of human activity, and indigenous organisms. Therefore, local habitats, home to microbes and animals including penguins, are excellent ecological systems to study the interactions between these different types of organisms. Over the past 50 years, the Antarctic Peninsula has experienced a major warming (Beyer, 2000; Vaughan et al., 2003; Steig et al., 2009), and resulting ice thaw has caused emergence of a large number of bare soil areas in summer (Pritchard et al., 2012; Nicolas et al., 2017). The expansion of the ice-free zone will lead to increased grounds for penguin activities and subsequently, penguin colony expansion (LaRue et al., 2013; Younger et al., 2015), although not an increase in penguin abundance. The combination of increased bare land areas and expanded penguin colonies will increase the likelihood of penguin affecting soil microbiomes.

Soil microbiomes are sensitive indicators of global change and integral part of biogeochemistry (Oyugi et al., 2006; Varin et al., 2012; Santamans et al., 2017), particularly the biogeochemistry of carbon and nitrogen (Kevin et al., 2014). The microbiome structure supporting its functions seems to be associated with an array of environmental factors in Antarctica, in a habit-specific manner. Some known factors include soil surface vegetation (Teixeira et al., 2010), organic carbon (OrC) (Tytgat et al., 2016), moisture (Lavian et al., 2001), and phosphorus (Chong et al., 2009; Roesch et al., 2012; Kim et al., 2014). Moreover, animal (penguins) activities, such as trampling and feces, have also been recognized to have a significant impact on soil microbial communities (Wang et al., 2015; Santamans et al., 2017). Animal feces could improve soil phosphorus (Zhu et al., 2014), organic matter, nitrogen (e.g., NO_3_^-^-N, and NO_2_^-^-N) (Legrand et al., 1998; Barrett et al., 2006; Aislabie et al., 2008). Besides, penguins excrete organic nitrogen (OrN) and ammonia into soils (Mizutani and Wada, 1988; Zhu et al., 2010; Crittenden et al., 2015). Continued release of these C and N resources to the exposed soils will result in changed soil geochemistry and microbiome composition. For example, many nitrogen cycles functional flora have been found in Antarctica (Tupas et al., 1994; Eckford et al., 2002). The availability of nitrogen will further drive the function of these nitrogen-utilizing flora and change the composition of the entire flora (Pochana and Keller, 1999; Guyonnet et al., 2017).

Here, we focused on the effects of penguin excreta on the exposed soils of the rookery grounds. We previously found that penguin rookery soils (ornithogenic soils) have different microbiome composition from pristine soils and soils colonized by human and seals (Wang et al., 2015). Santamans et al. (2017) showed Gentoo penguin and Chinstrap penguin are sea-to-land biotransporters of organic pollutants and trace metals which are released through fecal discharge into the soil and increase their content therein. Major change of microbiome components were identified to the level of class, which include Clostridiaceae and Bacillaceae of phylum Firmicutes and Actinomycetales of phylum Actinobacteria and reflect a direct role of penguin feces (Barbosa et al., 2016; Santamans et al., 2017) in microbiome composition of ornithogenic soils.

It is established that penguin feces change soil geochemistry which in turn affects the microbial composition (Santamans et al., 2017). Given this establishment, more detailed effects of penguin feces on important soil contents such as inorganic nitrogen and phosphorus can be further examined, and detailed changes in microbiomes between orthinogenic and non-orthinogenic soils need to be further characterized. Here, we hypothesized that besides direct inoculation of microbes, penguin feces can change orthinogenic soil geochemistry, particularly inorganic salts such as inorganic nitrogen, so as to impact microbial communities. To test this hypothesis, we selected a penguin rookery and its surrounding area in northern Ardley Island, Fildes Peninsula which is typic of maritime Antarctica. Samples were taken from one site in the middle of rookery and three sites from the surrounding area. To show the effects of penguin feces, all collected orthinogenic and non-orthinogenic soils did not contain feces. We measured the geochemical properties of the soil samples with nine metrics and analyzed bacterial composition using 16S rRNA gene. Based on these analyses, we further identified main geochemical factors shaping the bacterial community structure through redundancy analysis. Last, we used network analysis to extract modules of operational taxonomical units (OTUs), which were then correlated with each of the nine geochemical factors to determine the main shaping factors.

## Materials and Methods

### Study Sites and Sample Collection

**FIGURE 1:**
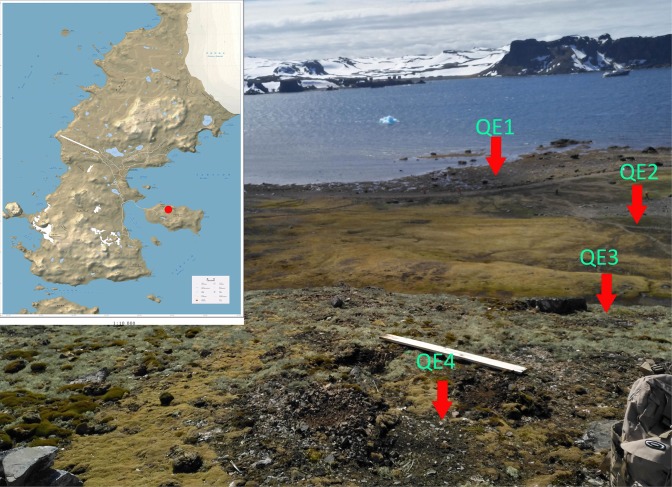
Locations of the sampling sites on Ardley Island, Fildes Peninsula in this study.

Four study sites (QE1, QE2, QE3, and QE4) on the coastal side of northern Ardley Island, Fildes Peninsula were selected (**Figure 1**). The main inhabitant of the island is the Adélie penguin (*Pygoscelis adeliae*). QE1 (62.21003°S, 58.93053°W) was below the penguin rookery; QE2 (62.21023°S, 58.92874°W) was in the middle of penguin rookery; QE3 (62.21124°S, 58.92878°W) was on the hillside at the edge of the penguin rookery; and QE4 (62.21123°S, 58.9293°W) was on the hill top without penguin activities. About 50 g of surface soil (0–5 cm) in triplicate was collected from each site using a sterile shovel and directly put into TWIRL’EM sterile sample bags (Labplas Inc., Sainte-Julie, QC, Canada). The samples were stored at -20°C in the Great Wall Station (China) for about 10 days before being transported in a cooler by air to the home laboratory for storage at -80°C until DNA extraction.

### Soil Geochemical Property Analyses

We analyzed nine soil geochemical properties: moisture content (MC), pH, OrC, OrN and five water-soluble nutrients including NH_4_^+^-N, NO_3_^-^-N, NO_2_^-_^N, PO_4_^3-_^P, and SiO_4_^2-^-Si (**Table 1**). Samples were dried in an oven at 105°C to constant mass to measure MC, which was determined as proportion of water loss from the wet soil wet (Schmugge et al., 2010). Soil pH was measured by adding 10 ml of distilled water to 4 g of soil and recording pH with a pH electrode (PHS-3C, Shanghai REX Instrument Factory, Shanghai, China). OrC and OrN were processed following Hu et al. (2012). The soils were freeze-dried and ground into powder, then treated with 10% HCl and dried to be analyzed on an element analyzer (EA3000, Euro Vector SpA, Milan, Italy). The soils used to determine nutrients were also freeze-dried and grounded, and then water was added at a ratio of 1:10 (g/mL). After shaking once every 4 h for 48 h, a nutrient auto-analyzer (QuAAtro, SEAL, Germany) relative standard deviation < 5% (Liu et al., 2016) was used to determine other physical and chemical properties.

**Table 1:** Geochemical properties of 12 samples investigated in the study.

Site	Sample	pH	Moisture content (MC) (%)	Organic nitrogen (OrN) (%)	Organic carbon (OrC) (%)	NH_4_^+^-N (μ g/g)	SiO_4_^2-^-Si (μ g/g)	NO_3_^-^-N (μ g/g)	NO_2_^-^-N (μ g/g)	PO_4_^3-^-P (μ g/g)
QE1	QE1.1	5.8	25.62	0.14	0.62	6.52	3.91	2.5	1.15	1.09
	QE1.2	5.91	31.34	0.31	2.26	15.6	9.36	5.99	2.76	2.62
	QE1.3	6.1	27.33	0.11	0.57	10.55	6.33	4.05	1.86	1.77
	Average	5.94 ± 0.15^a^	28.1 ± 2.94^ab^	0.187 ± 0.108^a^	1.15 ± 0.96^a^	10.89 ± 4.55^a^	6.53 ± 2.73^a^	4.18 ± 1.75^a^	1.92 ± 0.81^a^	1.83 ± 0.77^a^
QE2	QE2.1	5.5	66.22	1.24	12.05	82.49	49.5	31.68	14.57	13.84
	QE2.2	5.44	67.34	2.14	17.26	104.64	62.79	40.18	18.48	17.56
	QE2.3	6.04	66.59	1.08	10.21	99.77	59.86	38.31	17.62	16.74
	Average	5.66 ± 0.33	66.72 ± 0.57^acd^	1.487 ± 0.571^a^	13.17 ± 3.66^abc^	95.63 ± 11.64^abc^	57.38 ± 6.98^abc^	36.72 ± 4.47^abc^	16.89 ± 2.05^abc^	16.04 ± 1.95^abc^
QE3	QE3.1	6.05	18.15	0.12	0.76	3.19	1.92	1.23	0.56	0.54
	QE3.2	6.24	20.45	0.13	0.66	1.81	1.08	0.69	0.32	0.3
	QE3.3	6.25	18.35	0.13	0.68	1.77	1.06	0.68	0.31	0.3
	Average	6.18 ± 0.15^ab^	18.98 ± 1.27^bc^	0.127 ± 0.006^b^	0.70 ± 0.05^bd^	2.26 ± 0.81^b^	1.35 ± 0.49^b^	0.87 ± 0.31^b^	0.40 ± 0.14^b^	0.38 ± 0.14^b^
QE4	QE4.1	5.67	20.83	0.16	1.1	2.33	1.4	0.89	0.41	0.39
	QE4.2	5.65	17.23	0.16	0.91	2.87	1.72	1.07	0.49	0.47
	QE4.3	5.63	22.22	0.17	0.98	2.36	1.41	0.91	0.42	0.4
	Average	5.65 ± 0.02^b^	20.10 ± 2.58^d^	0.163 ± 0.006^b^	1.00 ± 0.10^cd^	2.52 ± 0.30^c^	1.51 ± 0.18^c^	0.96 ± 0.10^c^	0.44 ± 0.04^c^	0.42 ± 0.04^c^

*Between-sample difference indicated with lowercase superscripts (t-test, p < 0.05).*

### DNA Extraction and PCR Amplification

Genomic DNA was extracted from 0.25 g soil samples using MO BIO PowerSoil DNA Isolation Kit following manufacturer’s instructions. The purity and concentration of DNA extracts were detected on an agarose gel, and the qualified samples were selected for subsequent experiments. The v3-v4 region of 16S rRNA gene was amplified using primers 806R (′5-GGACTACNNGGGTATCTAAT-3′) and 341F (5′-CCTAYGGGRBGCASCAG-3′). All PCR reactions were carried out in 30 μL reactions, including 15 μL of Phusion^®^ High-Fidelity PCR Master Mix (New England Biolabs, Ipswich, MA, United States), 0.2 μM of forward and reverse primers and 10 ng template DNA. The PCR amplification cycle was: initial denaturation at 98°C for 1 min, followed by 30 cycles of denaturation at 98°C for 10 s, annealing at 50°C for 30 s, and elongation at 72°C for 30 s, with a final extension of 72°C for 5 min. PCR products were mixed with equal volume of 1X loading buffer (containing SYB green) and loaded onto 2% agarose gel for detection. Samples with a bright main strip between 400 and 450 bp were chosen for purification with Gene JET Gel Extraction Kit (Thermo Scientific, Waltham, MA, United States).

### Sequencing and Data Analysis

16S rRNA gene amplicons were sequenced on an Illumina MiSeq platform, and 250-bp paired-end reads were generated. Clean tag reads were obtained by removing barcode and primer sequences, trimming end bases and filtering low-quality bases. After quality control with Qiime (version 1.7.0) (Caporaso et al., 2010), chimeras were detected by (UCHIME Algorithm) (Edgar et al., 2011) and the Gold database, and finally the chimeric sequences were removed (Haas et al., 2011) to obtain effective Tags. The entire effective tags of all samples were clustered using the Uparse software (Version 7.0.1001) (Edgar, 2013), and the sequences were clustered into an OTU at a 97% identity. Meanwhile, the most frequent sequence for an OTU was selected as the representative sequence of the OTU. The species were annotated and analyzed on the representative sequence of the OTUs using Qiime and the SSU rRNA database (Quast et al., 2013) of SILVA (Wang et al., 2007) to obtain the taxonomic information and calculate abundance at each classification level in all the samples. Finally, all samples were normalized at the same sequence depth (51,249 reads), for the subsequent alpha and beta diversity analysis. The raw reads were deposited into the NCBI Sequence Read Archive (SRA) database (accession number: SRP120443).

Statistical analyses of the alpha diversity of soil samples, Chao1, Good’s coverage, ACE, and Shannon’s index (H’), were performed using Qiime software (version 1.7.0) (Caporaso et al., 2010). The species accumulation box-plot was plotted using the R software (version 3.2.4) (R Development Core Team, 2008) to check if the samples were sufficient. A linear discriminant analysis effect size (LEfSe) method was used to identify the significantly different bacterial taxa between sampling sites (Segata et al., 2011), which was also verified with one-way ANOVA followed by Tukey’s test. The relevance of environmental factors associated with the distribution patterns of bacterial communities of the samples was analyzed by Bray–Curtis distance-based redundancy analysis (db-RDA) using the R package vegan (Dixon, 2003). To find associations between geochemical parameters and specific microbial modules (subnetworks of OTUs), we used network analysis, following Guidi et al. (2016). Specifically, we first correlated each OTU with seven of nine geochemical parameters using sparse partial least square (sPLS) (Shen and Huang, 2008) as implemented in the R package mixOmics (Rohart et al., 2017). The global scale-free network of OTUs based on relative abundance (raised to the ninth power) from all the samples was constructed, and modules were identified using the R package WGCNA (Langfelder and Horvath, 2008). Modules, represented by the first principal component, were then correlated with geochemical parameters across all samples using Spearman correlation.

## Results

### Geochemical Properties of Soils Affected by Penguin Excreta

Our results showed penguin feces dramatically changed soil geochemistry of the rookery area compared to that of three other sites. First, the rookery (QE2) site has the highest values of eight of nine geochemical factors: MC, organic C, organic N, NH_4_^+^-N, NO_3_^-^-N, NO_2_^-^-N, PO_4_^3-^-P, and SiO_4_^2-^-Si (**Table 1**). Interestingly, the lowest values of NH_4_^+^-N, NO_3_^-^-N, NO_2_^-^-N, and SiO_4_^2-^-Si were detected at QE3. For example, the NH_4_^+^-N concentrations in the QE2 samples ranged from 82.49 to 104.64 μg/g, which was much higher than those in the other samples (1.77–15.60 μg/g), while the concentrations of SiO_4_^2-^-Si in the QE2 soils were from 49.50 to 62.79 μg/g, which were also much higher than those at the other sites (1.06–9.36 μg/g). Furthermore, we also observed some geochemical heterogeneity among the other three non-rookery sites. Specifically, six geochemical properties (MC, NH_4_^+^-N, SiO_4_^2-^-Si, NO_3_^-^-N, NO_2_^-^-N and PO_4_^3-^-P) were also different among the three surrounding sites (QE1, QE3, and QE4) (one-way ANOVA, *p* < 0.05). The values of the six geochemical factors at QE1 were higher than those at QE3 and QE4. For example, the PO_4_^3-^-P concentrations at QE1 (1.77–2.62 μg/g) was just lower than those at QE2 (13.84–17.56 μg/g), but higher than those at QE3 and QE4 (0.30–0.54 μg/g).

### Bacterial Diversity and Community Composition Changed by Penguin Feces

A total of 1,213,123 raw reads were obtained, which produced 785,534 Effective Tags after a series of treatments, averaging 65,461 valid sequences per sample. There are more than 1,500 OTUs in each sample; QE3.2 (the second sample of the site QE3) contains the most OTUs (2,734) and QE4.1 the least (1,592). The Good’s coverage estimator of the OTUs in the samples ranged from 0.990 to 0.996 (**Table 2**), indicating that the sequences sufficiently covered most of the bacterial diversity in all samples. The Shannon, Chao1, and AEC values at QE4.1 were the lowest among the 12 samples. The species accumulation box-plot (**Supplementary Figure S1**) saturates with all 12 samples, indicating the species diversity of the study sites should be well represented by these samples.

**Table 2:** Summary of the 12 samples in the present study.

Sample name	No. of clean tags	No. of effective tags	No. of OTUs	Shannon	Chao1	ACE	Good’s coverage
QE1.1	82,782	78,780	2,710	8.815	2,681.668	2,798.553	0.990
QE1.2	75,053	72,538	2,486	8.598	2,423.440	2,487.779	0.991
QE1.3	76,868	73,621	2,447	8.629	2,405.640	2,513.767	0.991
Average	78,234	74,980	2,548	8.681	2,503.583	2,600.033	0.991
QE2.1	60,284	55,877	2,212	8.314	2,173.320	2,211.856	0.993
QE2.2	69,581	63,223	2,381	8.612	2,336.016	2,346.260	0.992
QE2.3	69,046	64,234	2,476	8.390	2,451.780	2,500.181	0.991
Average	66,304	61,111	2,356	8.439	2,320.372	2,352.766	0.992
QE3.1	68,205	65,597	2,724	9.196	2,708.029	2,700.096	0.991
QE3.2	77,374	75,416	2,734	9.116	2,659.524	2,762.330	0.991
QE3.3	61,353	59,308	2,326	8.961	2,299.130	2,348.941	0.993
Average	68,977	66,774	2,595	9.091	2,555.561	2,603.789	0.992
QE4.1	56,092	55,203	1,592	7.912	1,467.725	1,528.680	0.996
QE4.2	68,107	64,624	2,611	9.154	2,565.547	2,594.912	0.992
QE4.3	60,340	57,113	2,584	9.056	2,554.120	2,567.884	0.992
average	61,513	58,980	2,262	8.707	2,195.797	2,230.492	0.993

**FIGURE 2:**
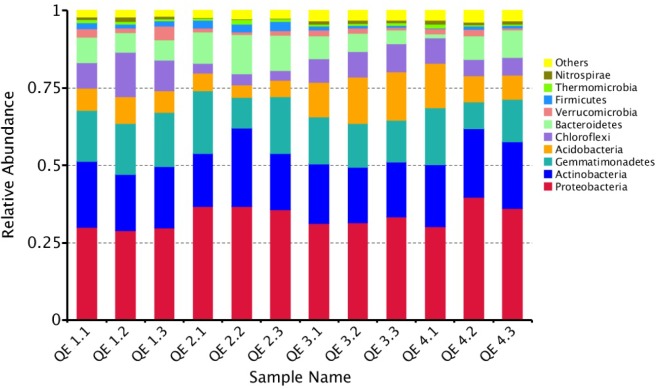
The top10 abundant phyla in the 12 soil samples in the study.

**FIGURE 3:**
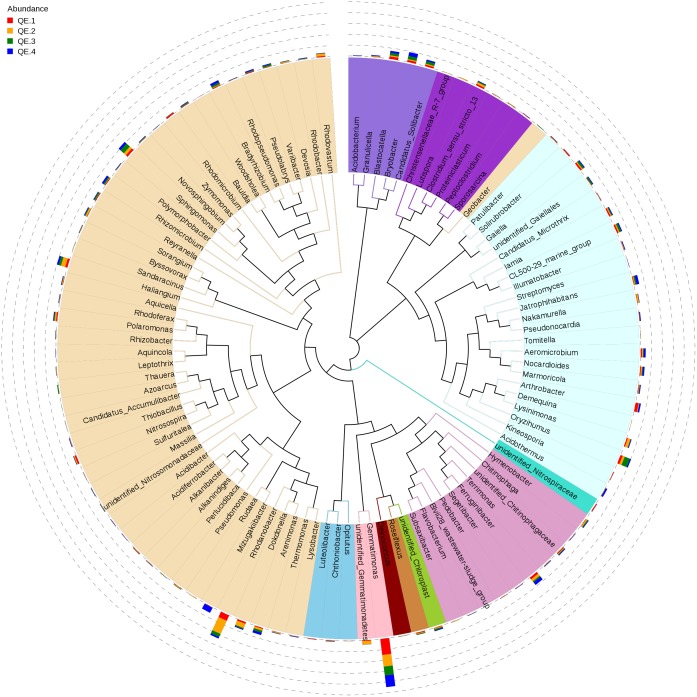
The phylogenetic tree of the top 100 genera. The colors of the branches represent the corresponding phyla. The outer bar plots represent the relative abundance of each genus.

To examine how penguin excreta affect soil microbiomes, we first looked at the general microbiome structure. Among the 12 samples, all OTUs are mapped to more than 40 phyla, the most enriched phyla based on relative abundance include Proteobacteria (33.36%), Actinobacteria (19.88%), Gemmatimonadetes (15.21%), Acidobacteria (9.17%), Bacteroidetes (7.56%), Chloroflexi (7.23%), Verrucomicrobia (1.77%), and Firmicutes (1.48%) (**Figure 2**). At the class level, the most abundant classes are: unidentified_Gemmatimonadetes (15.21%), unidentified_Actinobacteria (10.21%), Betaproteobac-teria (11.20%), Gammaproteobacteria (9.66%), and Alphaproteobacteria (8.49%). All these classes have more than 50,000 reads. Forty-nine of the top 100 genera (total reads > 269) belong in Proteobacteria, 25 Acidobacteria, and 10 Bacteroidetes (**Figure 3**). The most abundant is *Gemmatimonas* (32,949 reads, phylum Gemmatimonadetes), followed by *Rhodanobacter* (17,357 reads, phylum Proteobacteria), *Sphingomonas* (8,135 reads, phylum Proteobacteria), *Oryzihumus* (7,670 reads, phylum Actinobacteria) and *Haliangium* (7,359 reads, phylum Proteobacteria).

**FIGURE 4:**
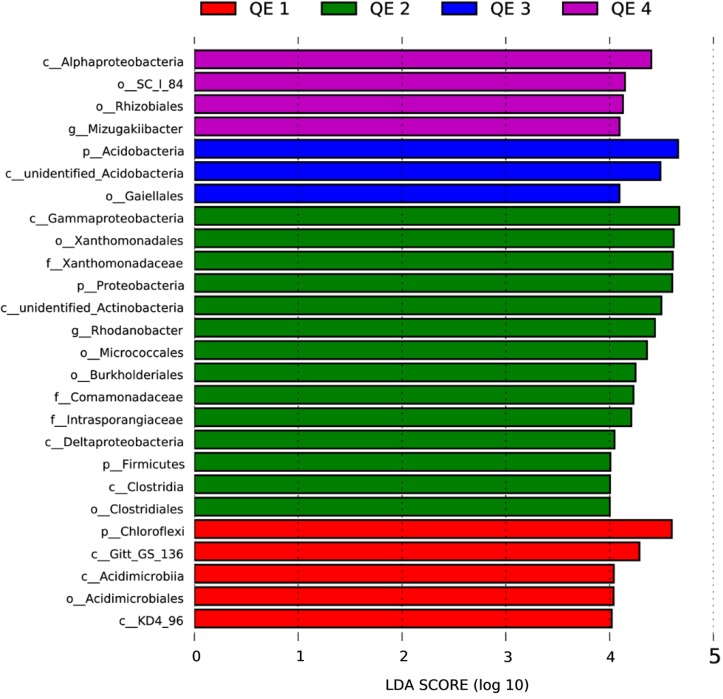
The LDA score distribution histogram is to search for Biomarker (Segata et al., 2011), which has a statistically significant difference between group and group, at all classification levels, and used the LDA score distribution histogram to show the species with LDA score larger than 4 in the present study.

Besides the overall community composition, we also compared the effects of penguin activities on the soil diversity. Based on the LEfSe results, 26 taxa showed LDA score (Linear Discriminant Analysis) greater than 4 (the cutoff for significance test) in the 12 samples (**Figure 4**). QE2 has most taxa, 14 of 26 (54%), more abundant than at other sites, which included classes, orders, and families of three phyla Proteobacteria, Firmicutes, and putative Acidobacteria. Interestingly, the phylum Firmicutes and its family Clostridiaceae are only enriched in QE2, which is generally of penguin origin (Barbosa et al., 2016; Santamans et al., 2017). Other sites usually have enriched taxa typical of soils, such as Acidobacteria at QE1 and QE3. Furthermore, we examined the dominant genera of Clostridiaceae and found two are highly enriched at QE2 than others: *Tissierella* and *Proteiniclasticum* (one-way ANOVA, *P* < 0.05; **Supplementary Table S1**). The abundance of *Proteiniclasticum* at QE2 is 2.5–9.9 times those at three other sites, and the abundance of *Tissierella* in QE2 is 1.7–13.6 times that of other sites. Another enriched non-Firmicutes genus is *Rudaea* (Gammaproteobacteria; Xanthomonadales; Rhodanobacteraceae) is extremely abundant at QE2 that its abundance is 7.0–22.5 times of that of other sites (one-way ANOVA, *P* < 0.05; **Supplementary Table S1**).

**FIGURE 5:**
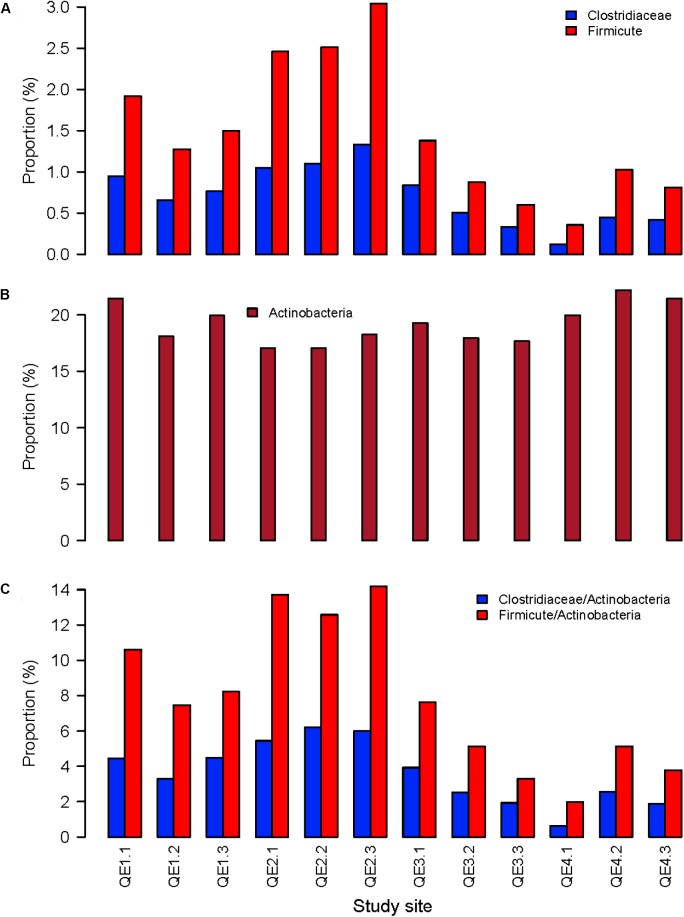
The relative abundance of the penguin-origin phylum Firmicutes **(A)** and its family Clostridiaceae and the soil phylum Actinobacteria **(B)**, and the ratios of Firmicutes and Clostridiaceae to Actinobacteria **(C)** the 12 samples from the four study sites.

**FIGURE 6:**
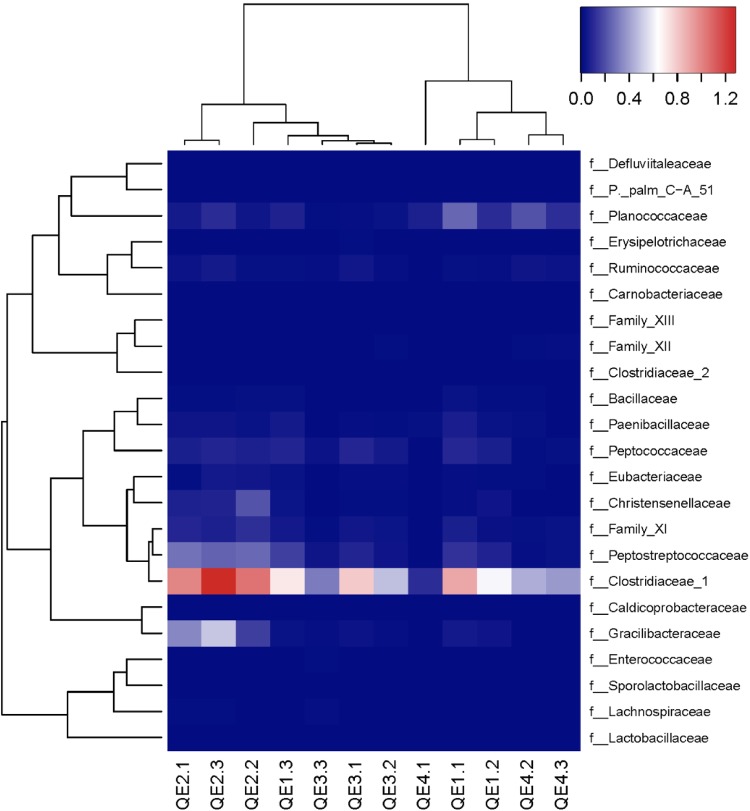
The families of Firmicutes of the 12 samples from the four study sites.

To further assess the effects of penguin feces on microbiome structure, we compared penguin-derived phylum Firmicutes and soil phylum Actinobacteria in abundance and composition. Consistent with LEfSe results, we found both Firmicutes and its order Clostridiaceae are significantly more abundant at QE2 than other sites, while Actinobacteria is not (**Figure 5**). Statistical results of one-way ANOVA with Tukey’s test are provided in **Supplementary Table S2**. In composition, there are 23 families of Firmicutes have been identified, including Clostridiaceae and Gracilibacteraceae, which are two most abundant of all families (**Figure 6**). Interestingly, some families in the QE2 samples form their own clusters based on abundance, and they are close to QE3 samples but well separated from QE1 and QE4 samples. Soil phylum Actinobacteria has 43 identified families, which also formed their cluster separated from three other sites (**Supplementary Figure S2**). Although Actinobacteria is not different among the sites in total abundance (**Figure 5** and **Supplementary Table S2**), there are some families such as Microbacteriaceae and Pseudonocardiaceae which are either more or less abundant at QE2 than other sites, respectively.

### Geochemical Parameters in Structuring Microbiome Composition

**FIGURE 7:**
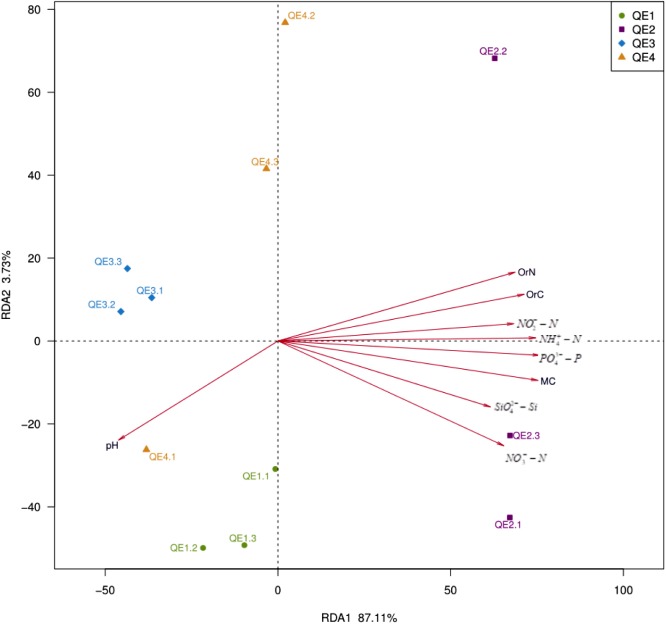
Distance-based redundancy analysis showing correlations between the bacterial communities and environmental factors of the 12 samples from the four study sites.

It has been established by the two previous sections that penguin feces change both soil geochemistry and microbiome structure. Here, we attempted to explore the association between them. First, we performed db-RDA analysis and found the 12 samples are well separated: QE2 is different from the other three sites along the first principal component RDA1 (**Figure 7** and **Table 3**). Interestingly, no two sites are close to each other; QE2 and QE4 have similar distribution along the second principal component RDA1, although no significant factors are included in this component (**Table 3**). All nine factors are included in the first component, with the effects of pH and the other eight appear to be in opposite directions, although pH is a component of RDA1, it was not significantly correlated with the overall microbiome composition (*r*^2^ = 0.39, *P* < 0.118). Based on the correlation with the first principal component RDA1 (**Table 3**), MC (*r*^2^ = 0.89, *P* < 0.001) was the most significantly correlated with the bacterial community composition in the study sites, followed by PO_4_^3-^-P (*r*^2^ = 0.88, *P* < 0.001) and NH_4_^+^-N (*r*^2^ = 0.85, *P* < 0.003), which are most similar in structuring these study sites and followed by OrC (*r*^2^ = 0.78, *P* < 0.005), NO_3_^-^-N (*r*^2^ = 0.77, *P* < 0.003), OrN (*r*^2^ = 0.74, *P* < 0.005), NO_2_^-^-N (*r*^2^ = 0.71, *P* < 0.005) and SiO_4_^2-^-Si (*r*^2^ = 0.63, *P* < 0.017).

**Table 3:** A Monte Carlo permutation test of relationship between environmental factors and bacterial community composition.

	RDA1	RDA2	*r*^2^	Pr (>*r*)	
MC	0.9867	-0.162551	0.8864	0.001	^∗∗∗^
pH	-0.912169	-0.409815	0.3898	0.118	NS
OrN	0.983909	0.17867	0.7409	0.005	^∗∗^
OrC	0.994916	0.100713	0.7808	0.005	^∗∗^
NH_4_^+^-N	0.999281	-0.037924	0.8479	0.003	^∗∗^
SiO_4_^2-^-Si	0.961419	-0.275087	0.6267	0.017	^∗^
NO_3_^-^-N	0.927653	-0.373443	0.7661	0.003	^∗∗^
NO_2_^-^-N	0.99995	0.010012	0.7091	0.005	^∗∗^
PO_4_^3-^-P	0.996001	-0.089347	0.8796	0.001	^∗∗∗^

*^∗^Correlation is significant at the 0.05 level. ^∗∗^Correlation is significant at the 0.01 level. ^∗∗∗^Correlation is significant at the 0.001 level. NS, not significant.*

**FIGURE 8:**
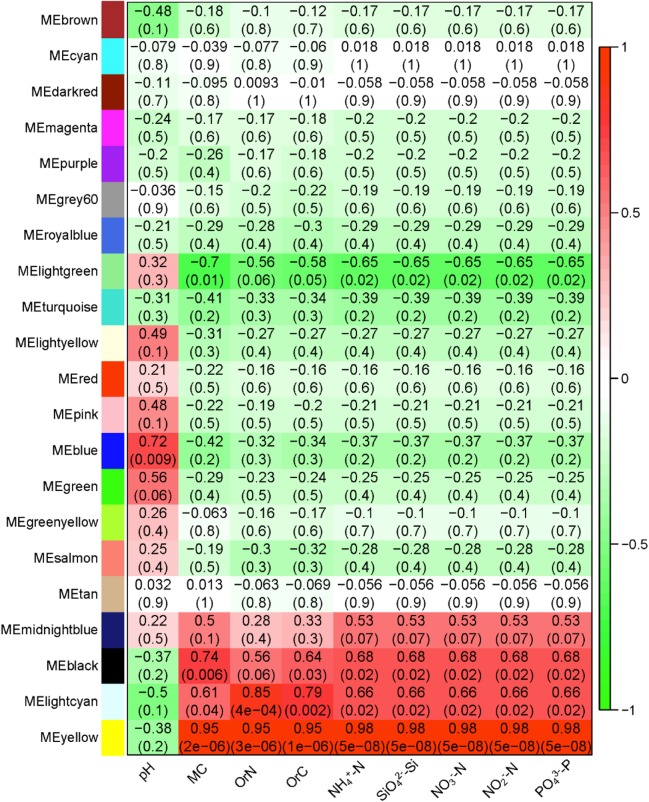
The correlation between the 21 identified modules with nine geochemical factors. The modules are indicated with colors as names. MC, moisture content; OrC, total organic carbon; OrN, total organic nitrogen. The red-to-green scale represents the correlation coefficients.

To further analyze the association between the microbiome and geochemical factors, we built a global co-occurrence network of OTUs and identified and extracted the modules of microbes which show high correlations with geochemical factors. A total of 21 modules which contain 4,318 of the 4,365 OTUs identified (**Supplementary Table S3**); the largest modules have 444 OTUs, and the smallest has 48 OTUs. These correlations confirmed the db-RDA results: the effects of pH and eight other factors are different (**Figure 8**). Specifically, among the 21 modules, three modules show strong positive correlation with all non-pH factors, and one shows a negative correlation; one module shows a positive correlation with pH. As MC is identified by RDA as most explanatory, we extracted the yellow module (**Figure 8**), these OTUs of this module indeed show high correlation between MC and module membership (**Supplementary Figure S3**). Among the OTUs of high degree (a metric of connectivity to immediate neighbors) and clustering coefficient are some *Clostridium* species, such as *Clostridium sensu stricto* and *Clostridium sordellii* and others in the family Clostridiaceae_1 of the order Clostridiales (**Supplementary Table S4**). Others hub taxa include g__*Rhodanobacter*, f__Chitinophagaceae, and g__*Gemmatimonas*.

## Discussion

Our study expands recent work which showed penguin feces have effects on microbiome composition of Antarctic ornithogenic soils (Santamans et al., 2017). Furthermore, we demonstrate the effects are both direct and indirect. Directly, penguin feces will change the soil microbiome composition through microbe loading, as observed by the presence of penguin-derived taxa (Barbosa et al., 2016) in ornithogenic soils, as previously observed (Wang et al., 2015; Santamans et al., 2017). Indirectly, the geochemistry of ornithogenic soils is very different from non-ornithogenic soils, as observed in other studies (Mizutani and Wada, 1988; Teixeira et al., 2010, 2013; Santamans et al., 2017). The correlations between different modules of soil microbes and geochemical factors are identified in this study.

We first observed in this study there is a direct effect of microbe loading to ornithogenic soils by penguin feces, as 23 families of Firmicutes of penguin origin (Barbosa et al., 2016), particularly Clostridiaceae is much more abundant in penguin rookeries than other sites. In contrast, typical soil phyla such as Actinobacteria shows comparable abundance between the rookery site and non-rookery sites. Although Firmicutes and Bacteroidetes consist of more than 90% of all phylogenetic types and are both dominant bacterial divisions in the animal’s gut (Leser et al., 2002; Eckburg et al., 2005; Ley et al., 2005; Ley, 2006; Barbosa et al., 2016), our results show Firmicutes is more abundant in ornithogenic soils in Antarctica, as found by Santamans et al. (2017). To provide further details on the family Clostridiaceae, we identified two genera, *Tissierella* and *Proteiniclasticum*, to be about 10-fold more abundant at the rookery than other sites. Besides Clostridiaceae of Firmicutes, we also found a non-Firmiculate *Rudaea* (Gammaproteobacteria; Xanthomonadales; Rhodanobacteraceae) being 15-fold more abundant in the rookery than other sites.

We also observed significant indirect effects of penguin feces on microbiome composition through soil geochemistry. These effects are most manifest in the study by Santamans et al. (2017), in which they find that OrC, OrN, and particularly trace metals are increased by penguin feces in Antarctica. Here, we showed the key inorganic nutrients, particularly elevated PO_4_^3-_^P, NH_4_^+^-N are from penguin feces. Most importantly, we identified 21 modules showing specific correlation with different geochemical factors. This highlights the differential nutrient needs of different taxa in the microbiome. High MC in the rookery site may be due to rich organic matter in the feces which alleviates soil weathering to improve soil water retention capacity (Harrysson Drotz et al., 2009). However, the relationship between soil moisture and organic content is complicated and often dependent on the timing of feces dropping being before and after soil thaw (Orchard and Corderoy, 1983; Wu et al., 2017). Among all explanatory factors, soil moisture is the main driver of soil C and N transformations in soils, because it affects microbial activity and survival, and a decrease in water content results in a decrease in the connectivity between the substrates and microorganisms (Chenu et al., 2014). Lavian et al. (2001) provided evidence that both activity and the composition and substrate utilization of the microbial community appeared to change substantially across the moisture level.

Besides the elevated content of PO_4_^3-^-P and NH_4_^+^-N, the high content of NO_3_^-^-N and NO_2_^-^-N may be due to nitrification from ammonia by some of the soil microbes. Coincidentally, the abundant *Rudaea* at the rookery site is capable of nitrification (Dong et al., 2016). On the other hand, pH and other geochemical factors have opposite effects in structuring the ornithogenic soil microbiomes. The role of pH has been found to be ubiquitously dominant in many types of microbiomes (Fierer and Jackson, 2006). One possibility for such a role is this may be a consequence of the increased fermentative metabolism linked to microaerophilic of anoxic conditions generated by the high oxygen demand associated with increased organic matter availability in penguin-affected soils (Lavian et al., 2001). Therefore, a functional analysis of these highly enriched taxa is needed to elucidate the process.

In this study, we found that penguin feces can impact ornithogenic soil microbiomes directly by microbe dropping and indirectly by changing soil geochemistry. Loading of microbes is manifest in Firmicutes and its class Clostridiaceae which contains two abundant genera. Penguins feces also cause changes in ornithogenic soil geochemistry, which has a high correlation with different modules of soil microbiomes. These altered microbiomes may in turns have significant functional effects on soil geochemistry structure in the rookery, which will be implicated in the biogeochemistry in maritime Antarctica.

## Author Contributions

NW planned the study, collected the samples, and conducted most of the lab work. YG and HC wrote the manuscript. GL, GR, JZ, YM, JL, and WH contributed to lab work and data analyses. YG and HC contributed to planning the project and data analysis. All authors reviewed the manuscript.

## Conflict of Interest Statement

The authors declare that the research was conducted in the absence of any commercial or financial relationships that could be construed as a potential conflict of interest.

## Supplementary material

FIGURE S1 The species accumulation box-plot of the 12 samples.

FIGURE S2 The relative abundance of families of Actinobacteria from the four study sites.

FIGURE S3 The correlation between OTU memberships in the yellow module of microbes with OTU significance for moisture content.

TABLE S1 One-way ANOVA for three highly abundant taxa in penguin rookery.

TABLE S2 One-way ANOVA and *Post hoc* Tukey’s test for taxa of penguin-feces origin or soil origin.

TABLE S3 The 21 modules identified among the 12 samples.

TABLE S4 The OTUs in the yellow module which shows high correlation with all the geochemical factors except pH.
