# Supplementary figures and images for: Direct and Indirect Effects of Penguin Feces on Microbiomes in Antarctic Ornithogenic Soils
Source: Front Microbiol. 2018 Apr 3;9:552. doi: 10.3389/fmicb.2018.00552 (PMC5891643; doi:10.3389/fmicb.2018.00552)

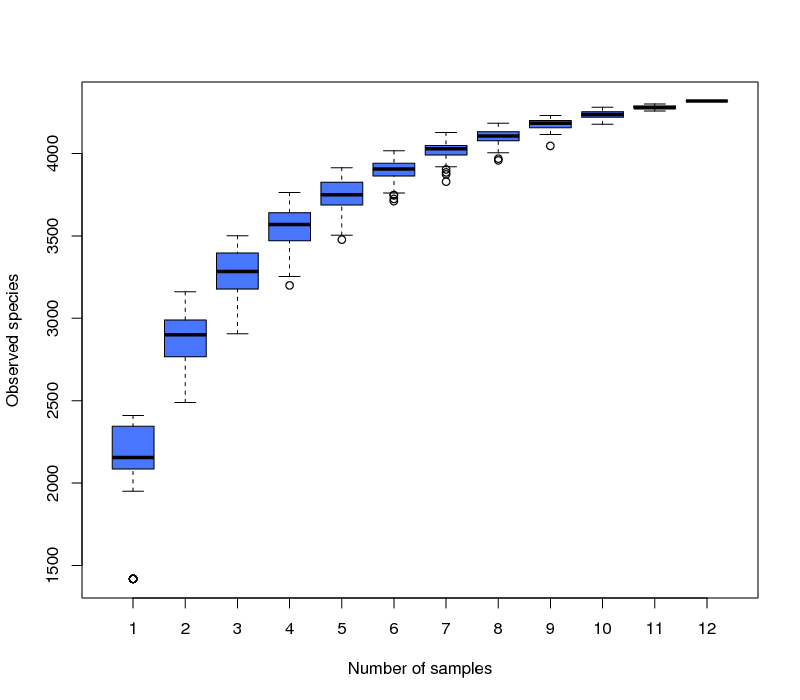

Supplement: FIGURE S1 — The species accumulation box-plot of the 12 samples. [file Image_1.tif]

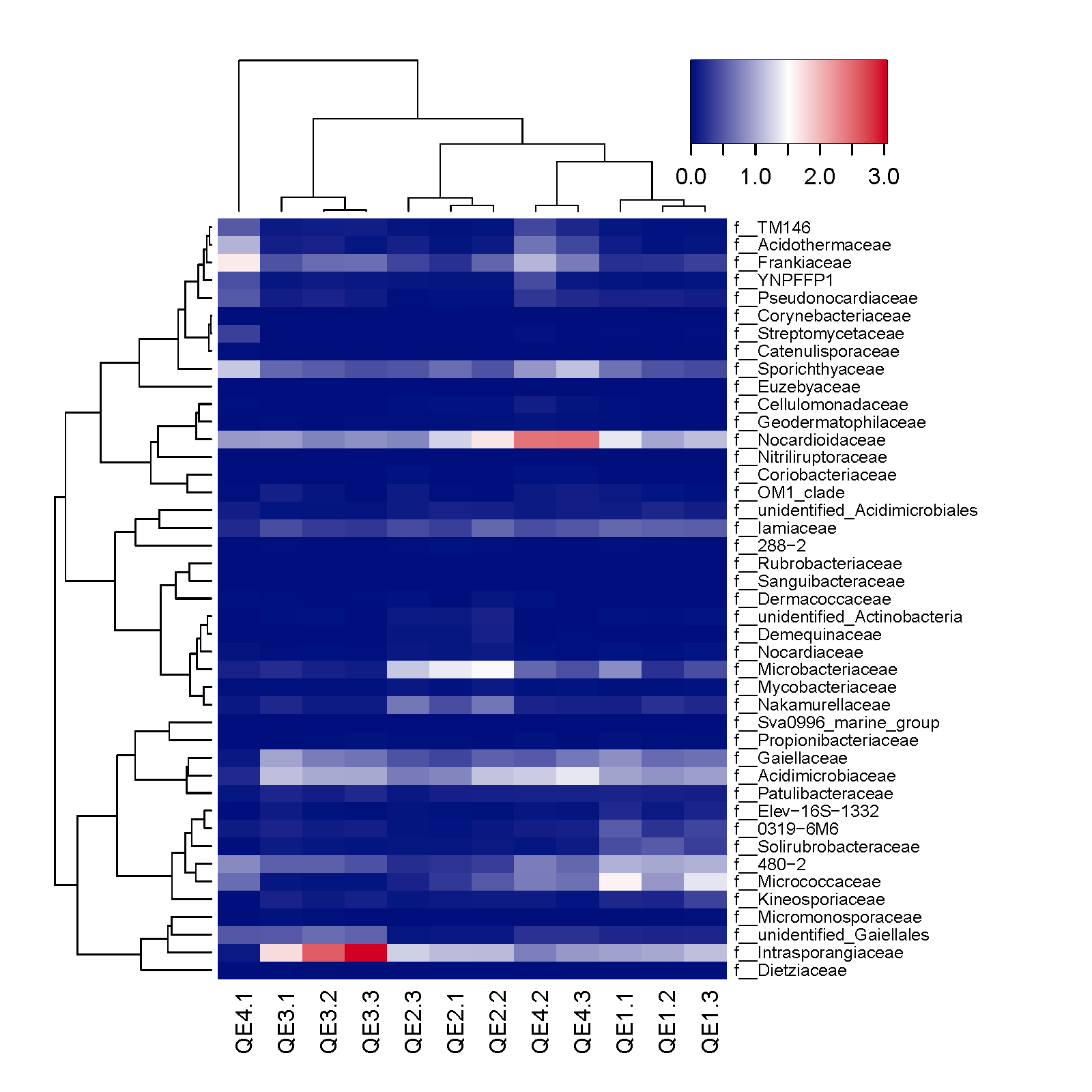

Supplement: FIGURE S2 — The relative abundance of families of Actinobacteria from the four study sites. [file Image_2.tiff]

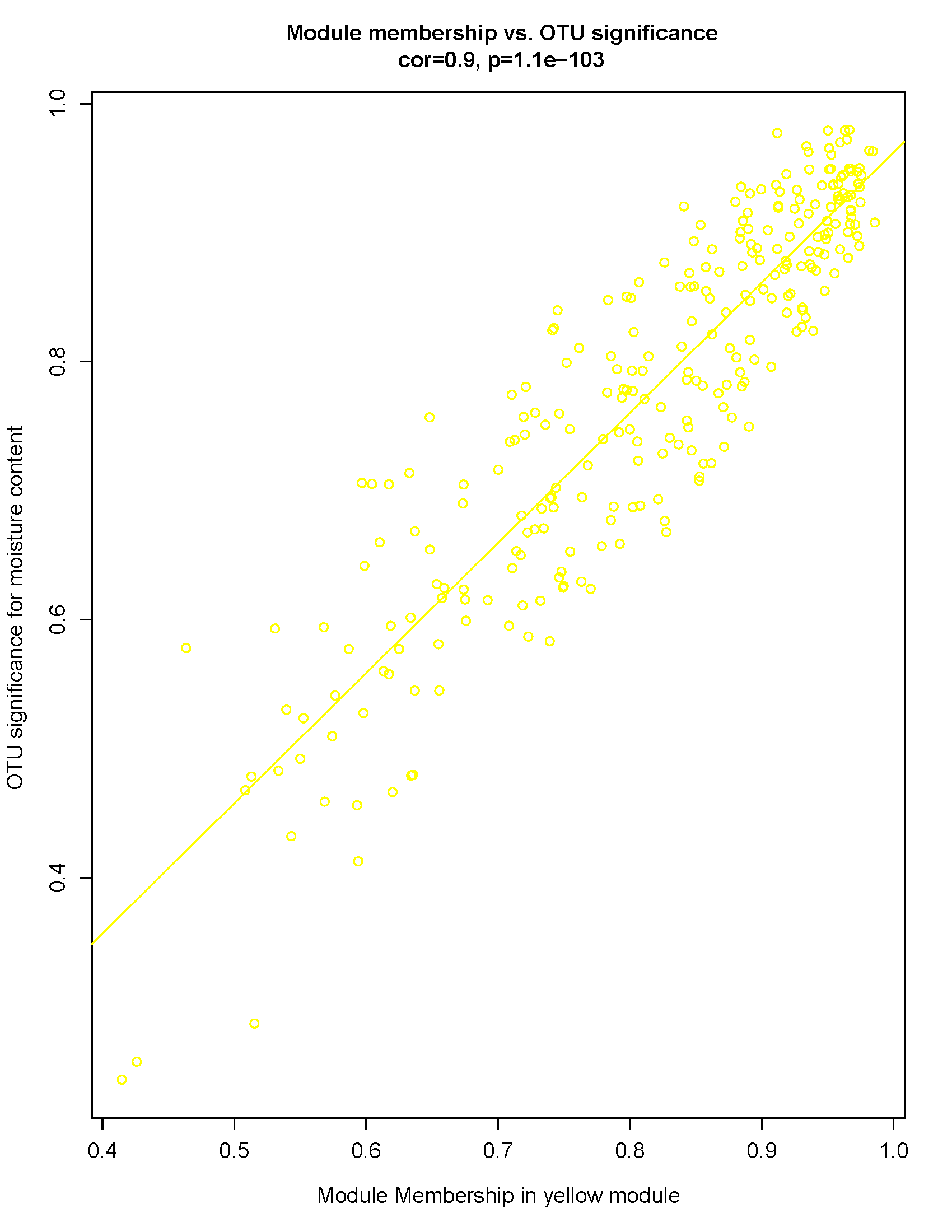

Supplement: FIGURE S3 — The correlation between OTU memberships in the yellow module of microbes with OTU significance for moisture content. [file Image_3.tiff]
